# Genome-Wide Localization of Protein-DNA Binding and Histone Modification by a Bayesian Change-Point Method with ChIP-seq Data

**DOI:** 10.1371/journal.pcbi.1002613

**Published:** 2012-07-26

**Authors:** Haipeng Xing, Yifan Mo, Will Liao, Michael Q. Zhang

**Affiliations:** 1 Applied Mathematics and Statistics, Stony Brook University, Stony Brook, New York, United States of America; 2 Computational Biology, Cold Spring Harbor Laboratory, Cold Spring Harbor, New York, United States of America; 3 Bioinformatics Division, Tsinghua University, Beijing, China; Ottawa University, Canada

## Abstract

Next-generation sequencing (NGS) technologies have matured considerably since their introduction and a focus has been placed on developing sophisticated analytical tools to deal with the amassing volumes of data. Chromatin immunoprecipitation sequencing (ChIP-seq), a major application of NGS, is a widely adopted technique for examining protein-DNA interactions and is commonly used to investigate epigenetic signatures of diffuse histone marks. These datasets have notoriously high variance and subtle levels of enrichment across large expanses, making them exceedingly difficult to define. Windows-based, heuristic models and finite-state hidden Markov models (HMMs) have been used with some success in analyzing ChIP-seq data but with lingering limitations. To improve the ability to detect broad regions of enrichment, we developed a stochastic Bayesian Change-Point (BCP) method, which addresses some of these unresolved issues. BCP makes use of recent advances in infinite-state HMMs by obtaining explicit formulas for posterior means of read densities. These posterior means can be used to categorize the genome into enriched and unenriched segments, as is customarily done, or examined for more detailed relationships since the underlying subpeaks are preserved rather than simplified into a binary classification. BCP performs a near exhaustive search of all possible change points between different posterior means at high-resolution to minimize the subjectivity of window sizes and is computationally efficient, due to a speed-up algorithm and the explicit formulas it employs. In the absence of a well-established “gold standard” for diffuse histone mark enrichment, we corroborated BCP's island detection accuracy and reproducibility using various forms of empirical evidence. We show that BCP is especially suited for analysis of diffuse histone ChIP-seq data but also effective in analyzing punctate transcription factor ChIP datasets, making it widely applicable for numerous experiment types.

## Author Summary

To unravel the mechanisms of gene regulation, understanding the complex interplay of protein-DNA interactions is instrumental. Recently, chromatin immunoprecipitation, coupled with next-generation sequencing (ChIP-seq), has risen as the go-to technique for examining these interactions on a genome-wide scale. It has also given rise to new computational issues. One such difficulty is the large variation in read density profiles from different types of NGS data, including variable peak “shapes” ranging from punctate to diffuse enrichment segments. To address this issue, we developed an infinite-state hidden Markov model that resulted in explicit formulas for the estimation of read density enrichment and can be used to find both significant “peaks” and broad segments. We show the versatility of BCP in analyzing various ChIP-seq data, which can further our understanding of the role of transcription factors in gene regulatory networks and histone modification marks in epigenomic modulation.

## Introduction

Recent technological innovations have transformed the study of DNA-binding proteins as higher throughput techniques have come to the fore. In particular, the widely used procedure involving in vivo immunoprecipitation of chromatin-bound proteins (ChIP) has benefited from significant innovation, undergoing several reincarnations, from ChIP-qPCR to ChIP-chip [1] and, most recently, to ChIP-seq [2], [3]. Capitalizing on the introduction of NGS technologies, ChIP-seq is being used to generate massive caches of data at an unprecedented rate [4]–[6]. Consequently, a bottleneck has manifested in our capacity to analyze this data. Developing practical tools for processing ChIP-seq results that are fast, accurate, and uniformly adoptable, is vital [7]. This is particularly apropos in light of the multi-institutional efforts that are underway, utilizing ChIP-seq to generate genome-wide profiles of chromatin-associated signals [8], .

ChIP has been commonly used for illuminating transcription factor binding sites (TFBS) [2], [3], but has more recently seen widespread adoption in studying epigenomic mechanisms—most notably, the role of post-translational, covalent histone modifications [4], [5], [10]. As a case in point, the NIH Roadmap Epigenomics Mapping Consortium has embarked on an effort to catalogue the most comprehensive database of epigenomic data to date—including data on over 25 histone marks, along with DNA methylation, chromatin accessibility, and small RNA expression [9]. Understanding the epigenome is crucial due to its purported involvement in myriad roles from individual diversity to development to cancer and other complex diseases [11]–[13]. At the molecular level, histone modifications, in particular, have been linked to regulation of transcription, gene silencing, and chromatin reorganization 12,14,15. These associations have given rise to the “histone code” hypothesis that could perhaps be a major mechanism for modulation of the epigenome [16].

ChIP can be broadly applied to study many protein-DNA interactions and on-going optimization is routinely introducing novel transcription factors and histone modifications to the diverse list of targeted proteins. From extremely sharp and punctate peaks to large, broad, and diffuse islands of enrichment, read profile signatures can span a wide range. Owing to this diversity, read profiles vary markedly and each presents its own nuanced challenges during downstream analysis. Algorithmically, punctate and diffuse enrichment have ostensibly been addressed as two mutually exclusive data types requiring distinct approaches. For instance, many transcription factors and histone acetylation modifications generate punctate profiles characterized by well-formed, sharply enriched peaks interspersed by large stretches of low signal. Several successful solutions have been introduced to address this problem [17]–[19]. However, as punctate peaks degenerate into more diffuse islands, read density enrichment appears far less pronounced, with much higher variance, and span much larger regions. In this scenario, peak-calling algorithms are extended beyond their intended scope and lose effectiveness [20]. Such non-punctate profiles are commonly observed when studying broad histone modifications, e.g. H3K27me3, H3K36me3, and H3K9me3. Instead, heuristic, window-based derivations have been developed to address this inadequacy [21], [22]. However, ambiguous, ad hoc parameters and compromised resolution have hampered widespread adoption of this class of island-detection tools. More recently, finite-state hidden Markov models (fHMMs), implementing the Baum-Welch algorithm or Markov chain Monte Carlo (MCMC) simulations, have been adopted to model diffuse read density profiles by classifying genomic regions into basal and enriched states [23]. The fHMMs usually focus on the broad enrichment data type spectrum by conceding the mutual exclusivity between detecting diffuse islands and punctate peaks and are used in addition to existing peak-callers. Therefore, consolidating the algorithmic landscape with a universal algorithm would have practical benefits by relaxing model assumptions on expected peak shape, size, frequency, or a mixture of these attributes [24].

Here, we introduce a Bayesian change-point (BCP) model that is based on recent advances in infinite-state hidden Markov modeling, as discussed by Lai and Xing [25]. Our model provides explicit formulas for the posterior means of ChIP-seq read density profiles and introduces a fast and computationally efficient approximation algorithm for estimating these posterior means. An enhanced signal is generated that can then be used to identify segments with a shared read density and the “change-points” that separate them. BCP enables analysis of whole genome ChIP-seq data with enhanced precision since read density estimates can adopt any real number value, providing added flexibility over HMMs assuming finite states. Furthermore, by virtue of the explicitly determined posterior means, a more detailed analysis of subpeaks within enriched regions can be interrogated. For example, recent work has suggested an exon-specific bias for H3K36me3 enrichment within gene bodies [26]. Therefore, BCP can quickly identify islands of histone enrichment that correlate well with known functional associations and are both reproducible and robust at high resolution. Additionally, BCP characterizes the diversity of ChIP-seq density profiles *in toto* and is easily adapted to segmenting sharper, punctate peaks with performance on par with a widely used peak-calling algorithm while maintaining proficiency in diffuse data types.

Our aim was to improve on existing methods for identifying diffuse histone modification enrichment by addressing some of the outstanding difficulties. We developed BCP to be fast and simple to use, minimizing subjectivity by requiring fewer user-defined parameters, and generating consistent results. We show that BCP advances diffuse, enriched-island detection and exhibits strong performance identifying peaks associated with transcription factor ChIP-seq data types.

## Results

### Algorithmic challenges of diffuse ChIP-seq data analysis

We sought to develop a method for reliably identifying large, diffuse regions of histone enrichment, an area of focus we felt could benefit from improved statistical models that more accurately capture the true nature of the data. Peak-calling algorithms often segment these broader domains into sub-peaks but fail to capture the more extensive context. We aimed to remedy this without undue reliance on ambiguous parameters while at the same time maintaining island continuity across regions of enrichment, independent of the input data type or usage settings. BCP models the ChIP-seq read counts data (**Methods**, “Data transformation”) using a Poisson distribution with a Gamma conjugate prior, which accounts for the inherent over-dispersion described in ChIP-seq data [27]. The parameters of the Gamma prior and the change point probability are estimated using an efficient method of moments search (**Methods**, “Hyperparameters estimation”). Similar to other HMMs, BCP takes into account the spatial structure of ChIP-seq data and attempts to identify change-points, positions separating two regions with different expected read depths (**Methods**, “Introduction”). In addition, we augmented computational speed with our Bounded Complexity Mixture approximation (**Text S1**, “Bounded Complexity Mixture (BCMIX) approximation”). BCP can perform a near exhaustive search for change-points in logarithmic time complexity, with only modest hardware requirements, making genome-wide analysis much more practically feasible.

**Figure 1:**
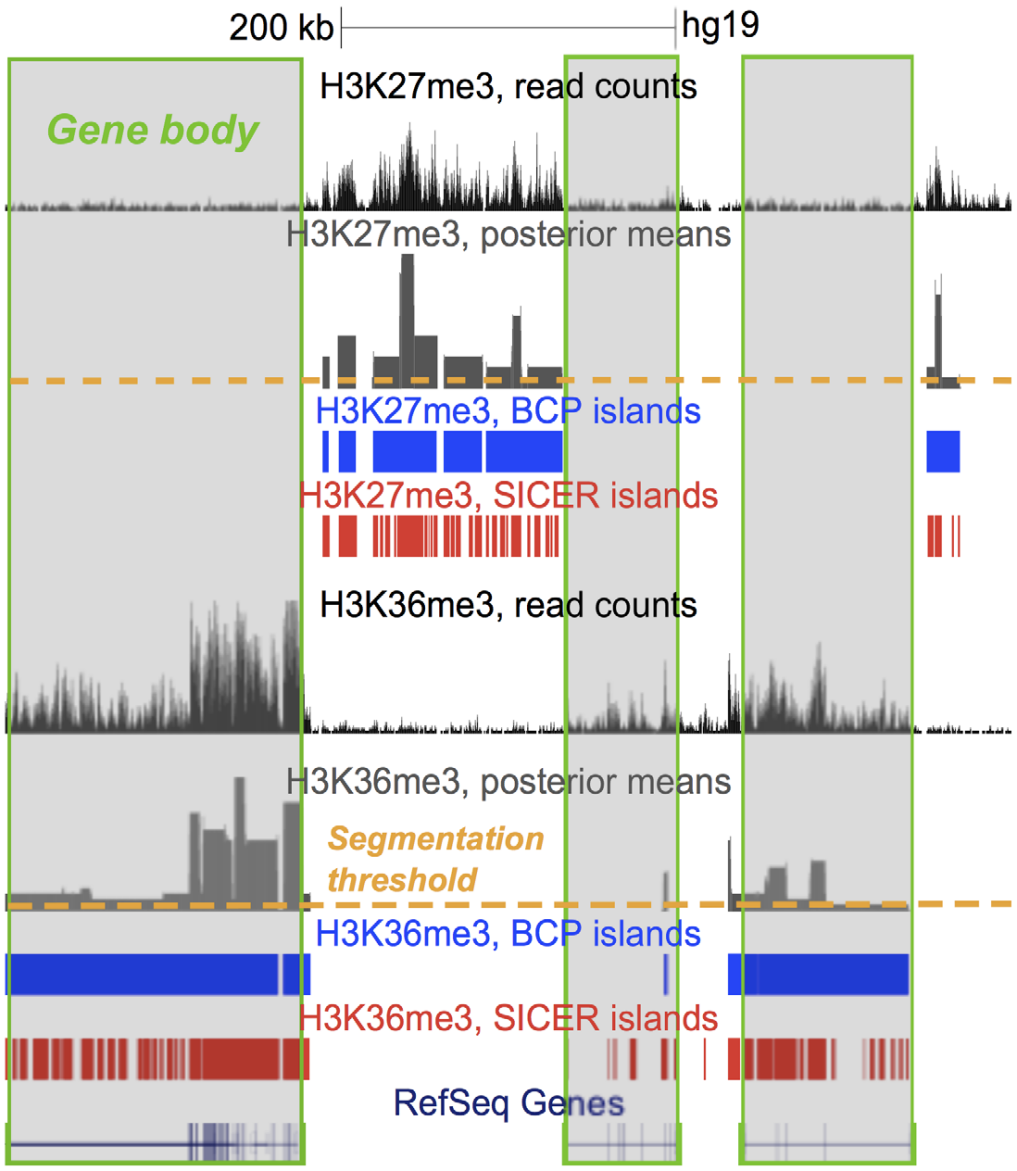
H3K27me3 and H3K36me3 diffuse histone marks. ChIP-seq was used to identify regions of enrichment based on read density profiles, visualized here in the UCSC genome browser (http://genome.ucsc.edu/). The enriched islands identified by BCP (blue) and SICER (red) are indicated. Additionally, posterior mean estimates used in BCP island detection are shown along with a line (orange) illustrating how thresholds are used to segment the signal. The correlation between H3K36me3 and gene bodies (outlined in green) and the mutually exclusivity of H3K27me3 and H3K36me3 were evident. The signal fluctuations caused by the highly variable read densities common to ChIP-seq data of diffuse marks is one of the notable difficulties for standard peak-calling algorithms, causing them to fragment the broader regions of enrichment into smaller, discontiguous peaks.

We used data from the Epigenomics Roadmap Consortium to illustrate some of these advantages [9]. These datasets were generated on the Illumina Genome Analyzer II platform at a read length of 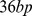 and were representative of most sequencing efforts. Our focus fell on the enrichment of the well characterized H3K27me3 and H3K36me3 modifications. H3K27me3 deposition confers gene silencing, often over large regions such as the entire Hox gene cluster [28]–[30]. In contrast, H3K36me3 has widely been associated with actively transcribed genes, and perhaps specifically exonic structures [4], [6], [31], [32]. These marks have islands that can span many tens of thousands of kilobase pairs (**Figure** 1). The propagation of H3K27me3 over large genomic distances has been well-documented [33], [34] and a similar phenomenon has been postulated more recently for H3K36me3 [35], [36]. These mechanisms are consistent with the notion of islands of enrichment present in each cell rather than an aggregated view of some varied mixture of placement in the population of cells. The broader islands resulting from the spread of histone marks, personifies the more complicated algorithmic task of identifying broad enrichment, thought to be distinct from sharp peak calling, that we hoped to address with BCP.

### BCP identifies H3K36me3 islands closely aligned to gene bodies

We tested BCP against SICER, which was in our consideration the most well rounded alternative for specifically identifying diffuse domains at the time of this study. Another viable method, BayesPeak [27], for example, required dividing each chromosome into smaller 6 Mb parallel jobs to run whole genome data efficiently, which we viewed as less than optimal. Transcription factor binding site prediction tools like MACS were excluded from this analysis since they were designed to address the punctate scenario and did not extend well to diffuse enrichment detection (“Table S1 and”Table S2 in **Text S1**). In order to objectively compare our BCP model to SICER's windows-based method, we reintroduced a metric called island read count coverage, or just island coverage, proposed by Zang, C., et al.(2009) [22], which, in brief, was defined as the number of reads falling within the boundaries of enriched islands divided by the total number of reads. We varied the different parameter combinations and compared both algorithms, keeping in mind the rationale that runs with similar island coverage can be considered comparable since this implies a similar extent of usage of the raw reads. We found that BCP islands in the H3K27me3 and H3K36me3 data sets routinely covered substantially more of the genome than SICER, despite similar island coverage, by virtue of appreciably larger island sizes, and more readily concatenated disjoined regions that were separated by low-density fluctuations in the read profiles. H3K27me3 islands were, on average, nearly 22.9 kb, called by BCP at an island coverage of 0.56, but only 4.2 kb for SICER, at a similar island coverage of 0.55 (“Table S1”in **Text S1**). This discrepancy was also observed in H3K36me3 islands. At an island coverage of 0.66, BCP's H3K36me3 islands were more than three times greater than SICER's—28.5 kb and 8.7 kb, respectively. Based on this, we concluded that BCP excelled at identifying large domain sizes expected of diffuse marks associated with clusters of repressed genes or actively transcribed gene bodies, as has been intimated for H3K27me3 and H3K36me3 [4], [28]–[32].

To address concerns we may have simply increased domain size indiscriminately, we validated our island calls using genomic features with known associations to the intensely studied mark, H3K36me3. Again, this covalent modification has been linked to gene bodies undergoing transcriptional elongation [26], so we reasoned that its related islands should correlate tightly with the boundaries of transcribed genes. We identified all RefSeq gene annotations (UCSC Table Browser, http://genome.ucsc.edu/) [37], [38] that intersected an H3K36me3 island, and determined, for each, how fully the gene was covered by an island. To accomplish this, we defined a metric, gene coverage, as the number of bases in a gene falling within an island call divided by the total number of bases in that gene and averaged this value across all overlapped genes. BCP showed reliably higher gene coverage versus SICER for all parameter permutations (**Table** 1). Furthermore, the fraction of genes covered, in BCP, over all parameters, was within a narrow range from 0.492 to 0.497, while SICER gene coverage ranged considerably more from 0.276 to 0.437. This suggested that BCP more precisely captured the gene bodies associated with H3K36me3 enrichment with less dependency on parameter selection.

**Table 1:** H3K36me3 islands and common associations.

	parameter	Avg. size^1^	gene coverage^2^	intergenic^3^	H3K27me3^4^	Rep.1 by 2^5^	Rep. 2 by 1^6^
	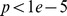	25.8	0.497	0.089	0.019	0.851	0.805
BCP^7^	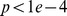	25.3	0.496	0.089	0.019	0.852	0.804
	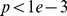	24.7	0.494	0.09	0.02	0.852	0.803
	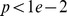	23.9	0.492	0.09	0.021	0.853	0.802
	W200-G200	2.7	0.323	0.085	0.021	0.689	0.805
	W200-G400	4.5	0.37	0.088	0.025	0.736	0.814
SICER^8^	W200-G800	8.7	0.437	0.094	0.032	0.8	0.818
	W400-G800	6.8	0.276	0.095	0.032	0.796	0.818
	W400-G1200	10.7	0.295	0.098	0.036	0.835	0.816

1 .the average island size in kb;

2 . the fraction of genes overlapped by an island;

3 . the fraction of islands covered by intergenic sequence;

4 .the fraction of islands overlapping H3K27me3 islands;

5 . the fraction of replicate 1 overlapped by replicate 2;

6 . the fraction of replicate 2 overlapped by replicate 1;

7 . island coverage: 0.66–0.67;

8 . island coverage: 0.62–0.68.

**Figure 2:**
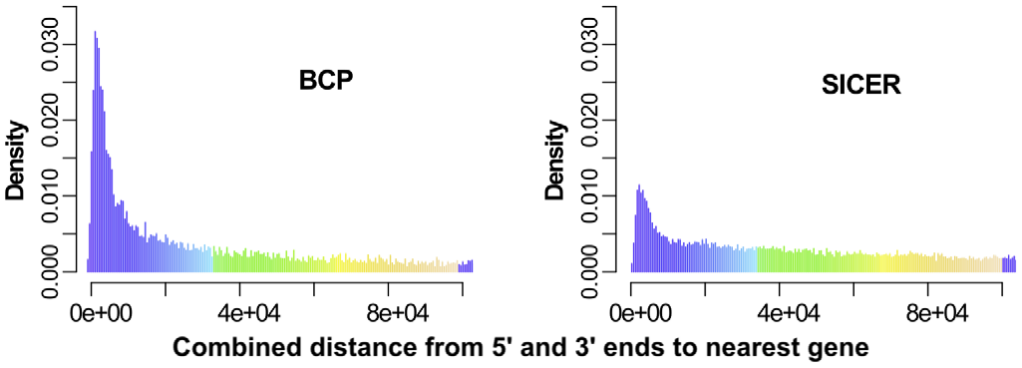
The distance from H3K36me3 island boundaries to nearest gene boundary was used as a measure of accuracy. H3K36me3 islands have been shown to correspond to actively transcribed gene bodies so we expected the boundaries of island and genes to coincide. The sum of the distances from both upstream and downstream island boundaries to the nearest gene boundaries were used as a per island error and illustrated in the histogram for BCP (left) and SICER (right).

To examine the proximity of islands to genes in more detail, we determined the distances from both upstream and downstream island boundaries to the nearest gene boundary. For simplicity, we only compared BCP using threshold 5 and SICER using a window size of 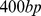 and a gap size of 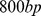 since, at these settings, their island coverage rates were similar—0.120 and 0.119, respectively (“Table S1” in **Text S1**). The sum of both these distances served as a measure of error, which we used to assess island detection accuracy. BCP islands had slightly smaller distances than SICER from the nearest gene boundaries, which is illustrated in the clear shift in the peak to smaller distances in the histogram shown in **Figure** 2 for BCP versus SICER.

### Improved gene coverage does not come at the expense of false positive rate

To certify the enhanced gene body coverage of BCP was the result of improved segmentation, we determined the empirical false positive rate by computing the fraction of identified islands overlapping intergenic space (UCSC Table Browser [37], http://genome.ucsc.edu/, Galaxy [39], [40], http://galaxy.psu.edu). Specifically, intergenic coverage was calculated as the number of bases in an H3K36me3 island, which overlapped any sequence defined as intergenic, divided by the total number of bases in the island, averaged across all islands. For the different run parameters, an intergenic coverage ranging from 0.089 to 0.090 of BCP islands was observed while a similar range from 0.085 to 0.098 of the SICER islands was observed (**Table** 1).

In contrast to H3K36me3, H3K27me3 is often associated with repression and commonly localizes to genes with little or no expression; in effect, it is anti-correlated with active transcription as has been described in previous genome-wide studies [4], [41], [42]. Furthermore, detailed analysis of chromatin states in plants by Hon, et al., demonstrated a nearly mutual exclusivity of these two marks [43] and a visual inspection of read density profiles suggested this was also the case in human data sets. Thus, we supposed that the presence of one of these marks should preclude the other and exploited this as a second false positive control for quantifying island validity. We again determined a simple overlap metric, H3K27me3 coverage, where we assessed the number of H3K36me3 island bases overlapping an H3K27me3 island and divided by the total bases in the H3K36me3 island. This was averaged across all H3K36me3 islands and reported (**Table** 1). Examining the H3K27me3 coverage, we found no glaring distinction between the fractions of SICER and BCP H3K36me3 islands overlapped by H3K27me3 islands; both fell within a similar range, from 0.021 to 0.036 and 0.019 to 0.021, respectively. Because of the similarity between the two methods in this comparison, coupled with the similarity in intergenic coverage, we concluded that the improvement in gene coverage was not the result of large, nonspecific island calls; BCP's advantage came without detrimentally impacting the false positive rate.

### Reproducibility and robustness

**Figure 3:**
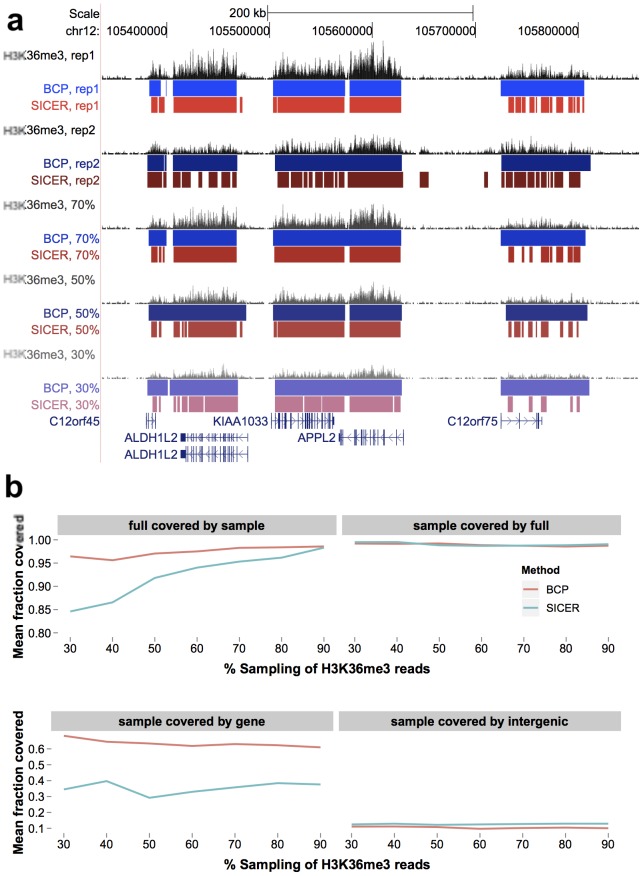
BCP was robust, providing consistent results in replicate and at various coverage depths. Using a second H3K36me3 data set and sub-samplings of the full replicate one dataset (30–90% randomly selected reads), we evaluated the reproducibility of BCP island calls. A) Enriched regions coinciding with gene coordinates were captured by the large, contiguous BCP islands (blue), while SICER islands (red) were more fractionated. B) We quantified the reproducible fraction of the full data set results versus the sub-samples (the number of full dataset island bases covered by a replicate/sub-sample island divided by total bases in full dataset islands, averaged across all islands) and vice versa. Also, we computed the fraction of island basepairs overlapping genic and intergenic regions (number of islands bases covered by genic/intergenic annotation divided by total bases in island, average across all islands).

To more definitively validate true positives, we obtained a replicate dataset of H3K36me3 from the Human Epigenomics Roadmap Consortium [9]. To supplement our analysis of genic/intergenic coverage, and H3K36me3/H3K27me3 anticorrelation, this added dataset was used to assess reproducibility. We defined legitimately enriched regions as those present in both replicates, and assessed the degree of overlap (again, the average, across all islands of one replicate, of the number of base pairs covered by an island from the opposing replicate divided by the total bases in the island). BCP islands exhibited a higher fraction of replicate 1 overlap by replicate 2 than SICER—ranging from 0.851 to 0.853 versus 0.689 to 0.835, respectively (**Table** 1). On the other hand, the overlap of replicate 2 by replicate 1 was surprisingly marginally higher for SICER than BCP—0.802 to 0.805 versus 0.805 to 0.816, respectively. This discrepancy appeared to be related to the respective read coverages of the replicates that led to an overall difference in island size. Replicate 2 had fewer reads (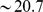 million uniquely mapped reads in replicate 1 versus 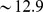 million in replicate 2) and lower coverage than replicate 1. As a result, SICER called replicate 2 islands that were subsets of the larger, more deeply covered replicate 1 islands (**Figure** 3a). We hypothesized that SICER was more sensitive to this coverage discrepancy than BCP, which managed to effectively extrapolate out the island boundaries, increasing average island size, despite the reduced read coverage. Presumably, our BCP model improved the ability to provide inference on true change-points across low-density “valleys by adjoining highly enriched regions through incorporation of spatial information. Such a feature would clearly be beneficial in the face of low or highly variable coverage between datasets. At first glance, the aforementioned incongruence of larger islands in lower density data hinted at poor performance. However, we suspected we might be observing a realization of BCP's theoretical advantage due to its more economical use of the read count information. In other words, despite fewer reads, successful island identification was still achieved and boundaries were reliably reproduced by BCP.

To substantiate this assertion, we sampled 30% to 90% of the full data set (replicate 1) by randomly selecting reads. Once more, we calculated a simple basepair-level overlap fraction—the average, across all islands in the full data set, of the number of basepairs in each full data set island, overlapping a sampled data set island, divided by the total bases in the full data set island. This overlap fraction represented a quantitative assessment of how reproducible the full data islands were at each of the sampling depths (**Figure** 3a). Even up to 30% sampling, BCP produced island calls consistent with an overlap fraction of at least 0.95 (**Figure** 3b, top left). In contrast, SICER dipped below 0.80 when analyzing the low sampled 30% data set. Of course, reproducibility must also be coupled to accuracy, so, to ensure this observation was not the result of indiscriminately large, non-specific islands, we reversed the comparison and determined the number of bases in each sampled data set island overlapping a full data set island, divided by the total bases in the sampled data set island, and averaged across all sampled data set islands (**Figure** 3b, top right). We found both algorithms maintained an overlap fraction around 0.98, which suggested no significant increase in false positive rate in BCP compared to SICER.

To demonstrate that BCP could achieve the same or better reproducibility and robustness against an objective marker, we compared islands to gene bodies, using the sampled data island calls, and calculated the fraction of each island covered by a gene. Even at the 30% sampling, we recapitulated—in fact exceeded—the coverage (0.68) observed in the 90% sampling set (0.61) (**Figure** 3b, bottom left). SICER coverage ranged from 0.29 to 0.40. We also calculated the intergenic coverage using the sampled data sets and found low coverage in BCP (0.097 to 0.11) versus SICER (0.12 to 0.13) (**Figure** 3b, bottom right). So, the improved gene coverage did not come at the expense of reduced specificity. Given these observations, we concluded that BCP provided a reproducible and robust determination of enriched islands that was consistently accurate, even in low coverage data.

### BCP is versatile

**Figure 4:**
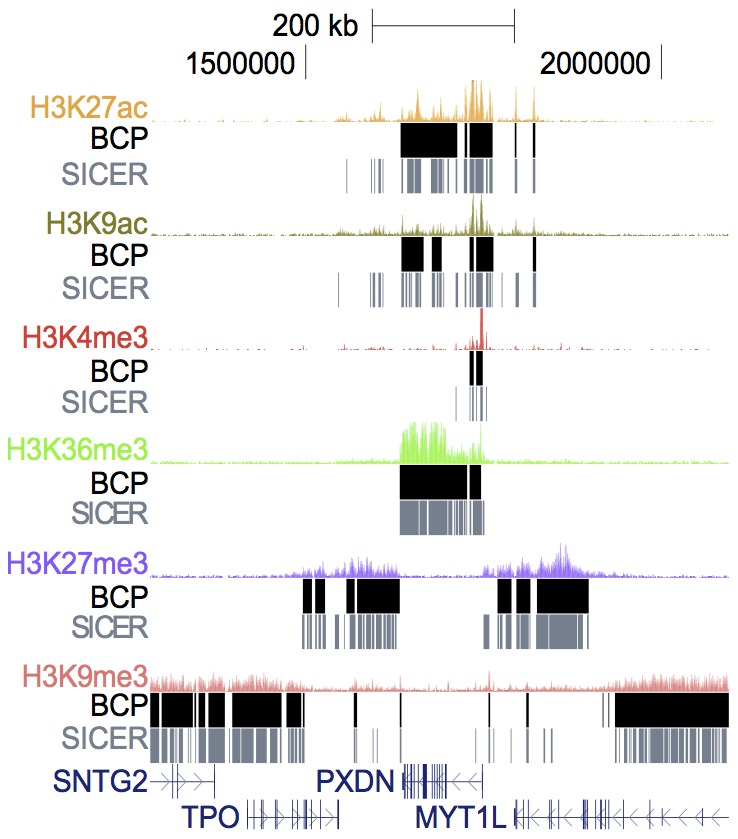
BCP dynamically adapted to many different types of data. To demonstrate its versatility, we compiled a set of several histone modifications and analyzed each under the default parameters for BCP and SICER. Regardless of the histone mark characteristics, whether more punctate as in acetylation marks and H3K4me3 or broad as in H3K27me3, H3K36me3, and H3K9me3, BCP (black) was able to make reasonable island calls that effectively described the underlying read profiles. SICER (grey) seemed more primed to identify smaller, sharper islands so often fragmented more general regions of enrichment.

One of our main concerns was avoiding tailoring BCP too specifically for the diffuse case, detracting from its effectiveness in punctate peak identification. For example, a generalized model for identifying large islands could be achieved by simply using arbitrarily large window sizes. Such a simplistic model might prove effective for some diffuse scenarios but would encounter difficulties in data comprised of smaller islands or sharp peaks. Consequently, we sought to develop a more versatile algorithm capable of handling various island sizes without precondition. To this end, we surveyed a wider complement of histone marks in the hopes of showing BCP was capable of analyzing each data set in this diverse collection irrespective of its read profile characteristics (**Figure** 4). We analyzed H3K27ac, H3K9ac, H3K9me3, and H3K4me3 data sets, to contrast BCP and SICER under default parameters, which highlighted BCP's versatility without tedious optimization. Qualitatively, BCP island calls captured the read density at least as well as SICER, which had a noticeably difficult time delineating broader islands. BCP was able to contract domain calls to widths expected of H3K4me3 enriched regions, which resemble punctate transcription factor binding sites. At the same time, BCP still managed to extrapolate larger domains to meet the broad diffuse size predicted of H3K27me3 domains. Default BCP was even able to identify notoriously troublesome, large, low-enrichment, H3K9me3 domains. This parameter-free performance could, in practice, circumvent the need for time-consuming parameter fine-tuning. Of note, even a single target protein dataset can be comprised of a mixture of both punctate and diffuse regions of enrichment, e.g., punctate H3K27me3 enrichment associated with bivalent promoter domains colocalizing with H3K4me3 enrichment along with the more common repressive, diffuse H3K27me3 domains. Both of these scenarios were successfully identified by BCP at high resolution with a single run without adjusting parameters.

### Transcription factor binding site detection

To test whether BCP was capable of evaluating other punctate ChIP targets, we analyzed NGS data previously generated on the Illumina Genome Analyzer, read length of 36 *bp*, from immunoprecipitation of the transcription factors CTCF [44] and STAT1 [3]. We applied the same general statistical model to calculate posterior means, effectively a smoothed representation of the raw reads. However, the nature of TF ChIP-seq data is significantly more punctate than in histone marks, so, we did apply a few modified preprocessing and post-processing steps (**Methods**, “Data Transformation”).

Since algorithms for identifying peaks in the punctate case have been thoroughly compared and contrasted [45], we chose one representative as a measuring stick to illustrate BCP's comparable performance, to make the case for its use for all ChIP-seq enrichment detection tasks. We chose MACS [17] as this representative since it has been cited extensively in ChIP-seq studies, is widely available as both source code and through the Galaxy software platform [39], [40], and has been shown to have accurate and efficient performance.

**Figure 5:**
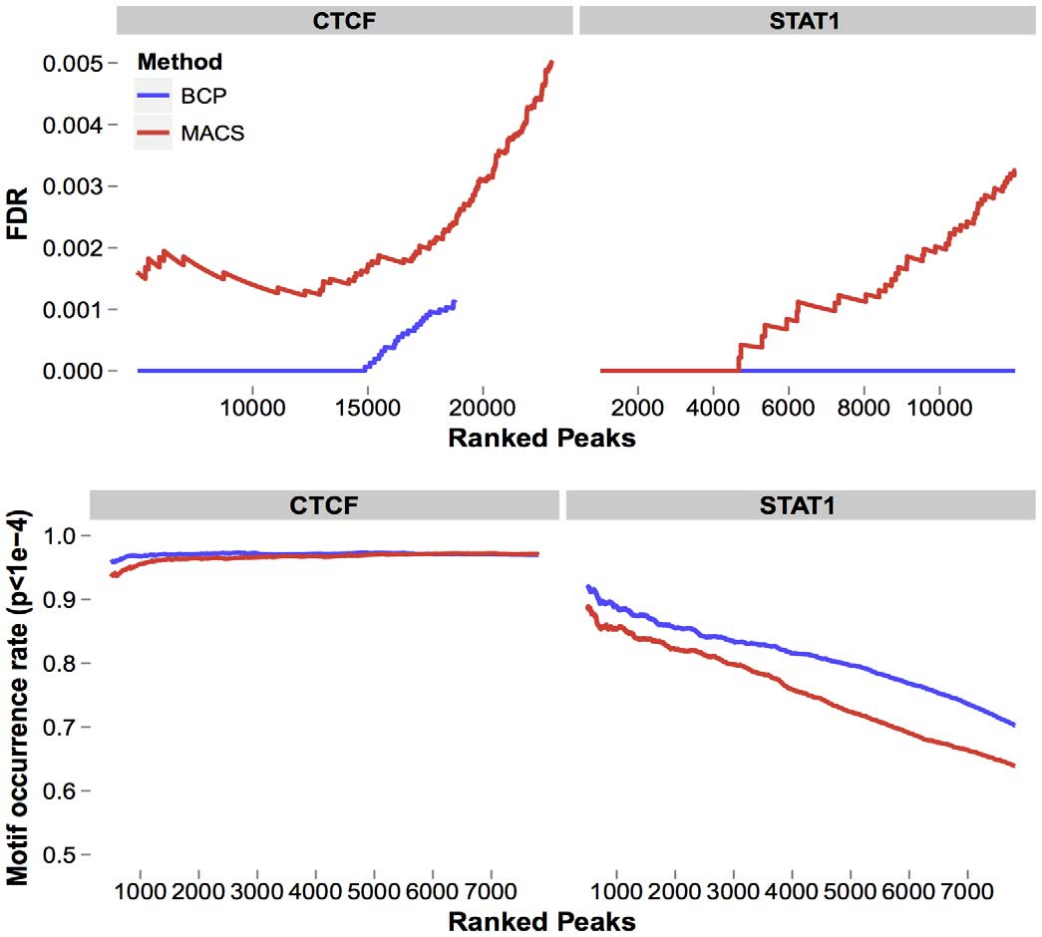
BCP showed strong performance in punctate transcription factor ChIP-seq data. Compared to MACS, a representative peak-calling algorithm designed for punctate peaks detection, BCP showed a comparable false-discovery rate (FDR) and rate of motif occurrence in both CTCF and STAT1 datasets. We apply the empirical FDR described in the Methods and by [17], dividing the negative peaks (detected when the input control sample was set as the test and the ChIP sample was set as the control) by the number of test peaks (the ChIP sample was set as the test and the input control sample was set as the control). Peaks are ranked according to p-value. Additionally, BCP displayed a slightly improved motif occurrence rate (the fraction of peaks containing a match to the TRANSFAC consensus motifs, as determined by STORM, 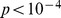).

The underlying biology of transcription factors is quite different than that of histone modification enrichment, so the same functional associations, like gene coverage, were not suitable. Instead, we chose more traditional metrics for assessing peak-calling performance. We evaluated the accuracy of peak calls, first, using an empirical false discovery rate as defined by Zhang, et al. (2008) [17]. This process entailed determining candidate peaks using the ChIP reads as the sample and the input reads as the control and, then, identifying “negative peaks” by inverting the read sets, using input reads as the sample and ChIP reads as the control. An empirical FDR was computed from the number of negative peaks divided by the number of candidate peaks. We also evaluated BCP and MACS using a second metric, motif occurrence rate. Using the published consensus position weight matrices for CTCF and STAT1 from the JASPAR [46] or TRANSFAC [47] databases, respectively, we searched the genome for significant matches (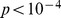) using STORM, part of the CREAD software suite [48], [49]. Sequences associated with each peak summit, 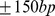-flanking regions, were iteratively scored as either with or without a motif match, in rank order according to the peak enrichment score. The cumulative rate of motif occurrence, the number of peaks with a match divided by the total number of iterated ranked peaks, was then plotted (**Figure** 5).

CTCF peaks were characterized by high signal-to-noise with little to no read density in between. Given such distinct peaks with so little intervening background, both algorithms easily identified peaks with a low FDR and high motif occurrence rate, perhaps with BCP exhibiting a slight advantage. STAT1 peaks were, in contrast, less refined and it is precisely in this scenario, where peaks degenerate to islands, that BCP excels so its advantages were highlighted; peak calls showed improved accuracy–with motif occurrence rates higher than MACS. Additionally, the FDR rate dropped more quickly to zero in the higher ranked peaks for BCP versus MACS. We concluded that BCP performance in punctate data was at least comparable, if not improved, over MACS, suggesting it can be a suitable tool for analyzing punctate ChIP-seq data.

## Discussion

Our main goal was to provide a novel solution for identifying islands of enrichment in diffuse data sets, principally diffuse histone modification data. As the selection of ChIP-seq “peak-callers” has become saturated, we hoped to introduce our offering as not just a niche supplement to a punctate peak caller but as a stand-alone solution to ChIP-seq data analysis, in general. Accordingly, achieving high fidelity in enriched domain identification in diffuse data without sacrificing performance in punctate data, and while preserving simplicity and ease-of-use, was paramount. Our BCP algorithm has several distinct advantages that we feel help it achieve this goal.

HMMs provide a natural model for finding read density change points using spatial information and have been applied widely with great success in genome research. Our model builds on this success by deriving explicit analytical formulas for infinitely possible states by calculating posterior means directly from read counts (**Methods**, “Data Transformation”).

In addition to this advance, we have made every effort to limit the number of user-defined parameters without affecting performance and reliability. BCP requires nominal user-defined parameters at runtime. The results are largely resistant to dramatic shifts when adjusting these parameters—relegating them mostly to fine-tuning—so, little time need be invested in parameter optimization. Fewer parameter permutations make variations between users, replicates, and experiments less problematic. In real world terms, coupling this with the explicit formulas and the BCMIX speedup algorithm presented an opportunity for considerable time savings. We compared BCP's runtime for whole genome analysis with SICER and MACS under identical hardware conditions on a high performance compute cluster at Cold Spring Harbor Laboratory (dual core 64-bit processors running at 2.0 GHz with 2GB of memory) using default parameters for each method. When studying histone modifications, such as H3K27me3 and H3K36me3, other algorithms took on the order of 4 to 5 hours, while BCP completion times averaged around 1 hour but optimally as short as 20 minutes. It took approximately 25 minutes for BCP to search for putative TFBS for CTCF and 50 minutes for STAT1. In contrast, MACS runtimes exceeded an hour when generating mappable wig/bedGraph tracks for visualization, as BCP does.

In the absence of a true “gold standard” for validating histone modification enrichment, we devised a cadre of simple metrics to characterize island accuracy. Namely, we investigated island coverage, correlation and anti-correlation with known associated annotations, reproducibility and robustness, and versatility. Based on existing literature, we operated on the assumption that ideal island designations should support the relationship between gene bodies and intergenic sequence compared to H3K36me3, the mutual exclusivity between H3K27me3 and H3K36me3, should be highly reproducible and robust across replicates regardless of coverage depth, and should be broadly applicable in light of the heterogeneity of histone modification data. BCP performed well in all of these indicators. While we do acknowledge their inherent weaknesses as individual performance metrics, we believe that, in aggregate, they represent a fair and comprehensive evaluation. So, in lieu of any “gold standard”, we believe our collective results show BCP has favorable performance and represents a strong candidate for analyzing diffuse histone modification data.

One noteworthy observation we have thus far left unaddressed is the apparent relationship between posterior means within regions of enrichment of H3K36me3 and exons, an association that has been previously described [26], [50]. Visual inspection of BCP's posterior mean estimates hints at a deeper relationship at a level of specificity beyond just actively transcribed gene bodies. The ability to interrogate this detail could represent a distinguishing property of BCP because of its unique capacity to directly calculate posterior mean estimates. Exploring this is part of our ongoing work, which we hope can add insight into the role of H3K36me3 in exon demarcation. In like manner, while we made every effort to address the known biases endemic to ChIP-seq experiments, e.g. amplification artifacts and ambiguous repetitive sequence (**Methods**, “overview”), some known, e.g. sequencing preferences for GC-rich regions or mappability differences, and perhaps some unknown biases still remain. Extensive research has been done along these lines to identify any systemic bias and other variability [51]–[53]. Furthermore, several bias corrections have been proposed [54]–[56] that could be incorporated into our model through an update to the empirical prior and further improve BCP's performance.

From a software perspective, we feel BCP's advantages serve an important purpose in improving usability without sacrificing fidelity. In a scientific climate that is becoming increasingly collaborative, an important precept was designing a method that would be readily standardized and whose results would be simply corroborated and easily applied over numerous experiments by multiple research groups. In this scenario, variability might come from several sources of experimental and technical noise, e.g., different end-users, technicians, or tissue culture and sample preparation conditions, inconsistent read coverage, inefficient sonication, inaccurate size-selection, etc [57]. Such fluctuations are endemic to ChIP-seq. BCP's Bayesian HMM underpinnings allow it to make inferences about this noise, in the context of spatial surroundings, leading to improved island continuity. In this regard, our results underscore the benefits of our model; BCP island calls remained robust, with reduced variability. Hence, the output regions should be readily comparable, with less concern over variability imposed by parameter choice, which we hope can facilitate collaborative efforts. Furthermore, the dependable island predictions should allow investigation of epigenomes in cell types and tissue contexts, without being restricted to relative genome coordinates, i.e., read densities gated on functional positions like transcription start sites, promoter regions, or distal enhancers [4], [58], [59]. We hope to leverage these advantages moving forward in future attempts to make novel discoveries with regards to epigenetic regulation.

## Methods

### Overview

BCP accepts the browser extensible data (BED) format (UCSC genome browser, http://genome.ucsc.edu/), which we transformed to read counts at every genomic location for each chromosome. Only reads mapping to a unique genomic location were considered and only a single read per start/end coordinate was allowed to reduce spurious amplification and repetitive sequence bias. In the case of transcription factor ChIP data, adjacent positions with identical read counts were “blocked” together. For histone modification ChIP data, read counts at 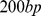 adjacent windows were calculated (**Methods**, “Data Transformation”). This window size is the default setting for BCP and was chosen for two reasons. First, a single nucleosome is the expected smallest unit size for histone modification data, including wound and linker DNA, and is roughly this length. Second, 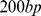 is approximately the size-selected length, following DNA fragmentation, for most library preparation protocols. The user can adjust the window size, but in our experience, optimization away from the default value was rarely necessary. We assumed that read counts or average read counts on within “blocks” or windows, respectively, follow a Poisson distribution with mean 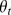, 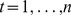, where 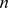 is the number of “blocks” or windows in the chromosome, and the true signal 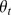 may undergo occasional change with probability 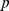 at each location 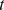. We also assume that when 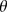 changes to a new value at 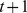, the new value follows a 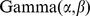 distribution. Under this setup, the posterior distribution of 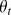 given all the data is a mixture of Gamma distributions (**Methods**, “Model specification”),
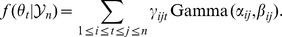



Hence 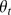 can be estimated by a weighted average of posterior means with different window sizes. In practical analysis, the model parameters 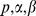 can be replaced by their maximum likelihood estimates, and the mixture above can be approximated by a bounded complexity mixture (BCMIX) algorithm (**Text S1** “Bounded Complexity Mixture (BCMIX) Approximation”).

BCP, as a change point model, has key differences with other similarly minded methods. Its estimate of true signal requires no prior knowledge of the number of different states of 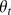, nor the positions or magnitude of the change points. The posterior mean, as an estimator, plays an important role in peak calling (TFBS) and/or segmentation (HM) and we implemented it directly to finding putative TFBS and histone-mark enriched islands. Given the posterior mean of each block or window represents a piecewise constant signal, smoothed by incorporating upstream and downstream information, “false” enrichment areas caused by local noise were minimized and our ability to identify the most likely enriched region was enhanced. Consequently, “gaps” in large significant domains were marginalized and we performed segmentation using a simple cut line across the posterior means decided from the background signal (**Methods**, “Peak calling and segmentation”). After generating candidate segments, each was substantiated as a peak or island of enrichment if the number of ChIP reads within the region surpassed the 90th-quantile value expected assuming read number follows a Poisson distribution with a mean based on the number of input reads in the same region.

### Data transformation

Before applying our model, the small reads sequenced from the DNA fragments were processed depending on the ChIP experiment protein target, either TFBS or histone modification.

For TFBS studies, highly-enriched binding sites among relatively low background were singled out. Given the bimodal profile of raw read distribution between plus and minus strands, true TFBS were more likely located centrally between a plus and minus peak; so, we first paired highly enriched local maxima from both strands to estimate the shift size. The small reads were then shifted towards the center, which put the mode of the read density at the center of each fragment. This represented the most probable location of each TF binding. We then transfered the read coordinate data (“BED format”) into read counts data, the number of reads overlapping each position. Because such aggregate read counts were intrinsically piecewise constant along a chromosome, we considered each piecewise constant fragment as a block and denote 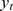 as the common read count of block, 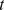. Notably, the sizes of the blocks were often different and represented data-driven window-size selection.(“Figure S1” in **Text S1**)

For histone modification analysis, since the purpose was to distinguish enriched segments only a few fold greater than background with highly variant signals, we extended the reads to a user-specified fragment length, and then calculated the read counts for each position as in the TF case. We then partitioned the read count sequence sequentially as consecutive “blocks” with block size 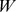 and let 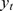 be the average read count in block 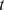 (we round 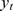 to the nearest integer). In our analysis, we choose 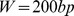, which is the approximate length of a single nucleosome unit (“Figure S2” in **Text S1**).

### Model specification

Let 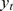 be the read count in the 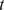
th block, where 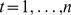. The way of obtaining 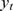 depends on the experiment (**Methods**, “Data Transformation”). Our goal was to find either peaks of TF binding or identify enrichment regions in histone marks. Assuming the transformed data 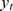 followed a Poisson distribution with parameter 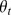 on each block, where 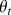 represents the mean of 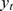 in each block (different from integer 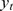, 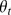 can be fractions). Given 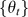, 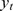 are independent. 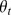 is piecewise constant and the indicators 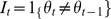 are independent and identically distributed Bernoulli random variables with success probability 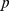. When 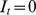, 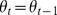; otherwise, the numerical value of 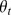 move to another level which follows a 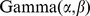, which is the prior conjugate distribution and accounts for the long-tailed over-dispersion underlying the data. Note that in contrast to most HMMs previously reported, which assume a finite number of values (or discrete state space) for 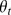, we allow an infinite number of values (or continuous state space) for 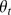.

Denote 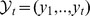, 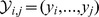, and 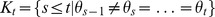 the most recent change-point before or equal to the 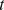
th block. Then given 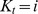, the posterior distribution of 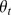 is 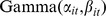, in which 
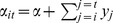
 and 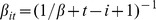. Letting 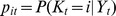 and denoting 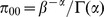 and 
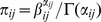
, one can show that the posterior distribution of 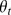 as
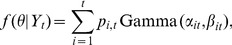
(1)in which 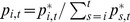 and
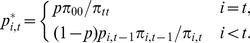
(2)


Similarly, the location-reversed counterpart is obtained as follows:
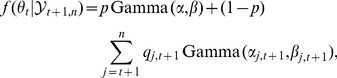
(3)where 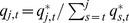 and
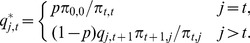
(4)


Applying Bayes' theorem to combine (1) and (3) yields the posterior distribution of 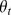 given 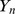.
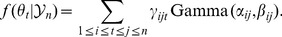
(5)where 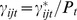, 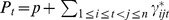 and
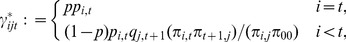
(6)


The above distribution yields the estimate of 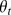


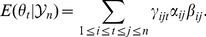
(7)


The estimate (7) can be considered a dynamically-adjusted “scan statistic”. However, it is distinct from classic scan statistics for two reasons. First, no window size is specified in the estimation procedure; all window sizes are considered with different weights, 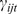. Second, the possibility of a change-point in 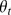 is incorporated into the calculation of the weight, in conrast to classic (weighted) scan statistics that are not constructed based on nonlinear features of the data.

### Hyperparameters estimation

The Bayes estimates 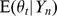 involve the hyperparameters 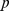, 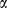, and 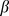, which are replaced by their estimates in the empirical Bayes approach. From the definition of 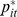 and (1), it follows that the likelihood function of 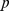, 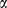, and 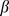 is
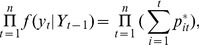
(8)in which 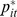 is a function of 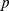, 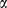 and 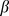 given by (2). Since the 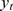 are exchangeable random variables in our model, we can estimate 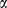 and 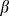 by the method of moments. The important hyperparameters in the change-point model are the relative frequency 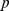 of change-points. Putting the estimated 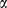 and 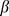 into (8), we can estimate 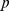 by maximizing the log-likelihood function 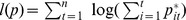, which can be conveniently computed by grid search [25]. Notice the log-likelihood function above also has an explicit formula and the a which we use to search for p, which has the form 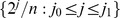, where 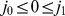 are integers. So the parameter estimation is very efficient despite the large scale of the data. In practical analysis of ChIP-seq data, as the number of change-points are much smaller compared to the sample size, one can simply use values that are close to 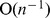.

### Peak calling (TFBS) and segmentation (HM)

The above discussion on the estimation of 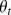 (**Methods**, “ Model specification”) indicates that our estimation procedure is purely data driven and incorporates the spatial structure of data. We now discuss the post-analysis of our model.

In searching for peaks in TFBS, we only consider areas on which 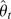 were larger than a certain threshold, 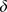. Since most genomic regions only contained background signal, we chose 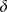 to be the 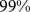 quantile of Poisson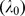, where 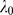 was the average of all read counts 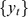. We then found the block within each area in which 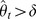, with biggest posterior mean and extended in both directions if the difference of adjacent posterior means was less than one. The extended areas were considered an approximation of the true enrichment area. We called this a sub-area within the enriched peak candidate and the position within the peak candidate with largest posterior mean was called the summit. (“Figure S3” in **Text S1**) As there were many factors in a ChIP experiment that can lead to false positives, input (control) data sets were used to filter false candidates. For each candidate peak, we used a window the size of the candidate peak and extended the summit of each candidate peak by a distance ranging from the length of one-window to five-windows. We also determined the average number of input reads in these extended areas versus the sub-area without extension in the input (control) data and chose the larger of the two as 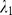. The average number of reads in the ChIP-seq data was calculated for the peak candidate area as 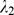. We then performed a simple hypothesis test for each peak candidate with the null hypothesis that 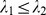 and rejected the 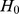 with some small p-value which indicated that 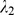 was significantly enriched over input background.

A similar process for segmentation was applied to study HM marks. Since HM data were more diffused, we used a more lenient threshold 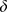 (we chose 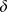 to be the 90% quantile of Poisson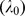, where 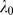 was the average of all read counts 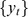). Since the posterior mean was a smoothed read density forming an approximately piecewise constant profile (“Figure S4” in **Text S1**), those segments with posterior mean greater than the threshold gave us candidate segments, in which we then filtered out false positives by using input (control) data. As the segments were broader than in the TF ChIP-seq data, it was not necessary to apply the window extension step to account for local background variation flanking candidate regions. Hence, we simply screened each candidate region using the average number of reads within the enriched region for ChIP and input samples and applied a hypothesis test, as before.

### Quantifying performance

In general terms, the islands identified in this study were compared to some other feature, whether it be gene bodies, intergenic space, the replicate, or sub-samples, as follows. The islands that did not intersect the feature of interest over at least one base pair were first filtered out. For each remaining island, the number of base pairs intersecting the feature of interest was divided by the total base pair length of the island itself, giving its overlap ratio. The ratios of all remaining islands were averaged to give the final values reported in **Table** 1 and plotted in **Figure** 3b.

The empirical FDR used to evaluate performance in analysis of transcription factor ChIP-seq data sets, CTCF and STAT1, was, again, adopted from Zhang, Y. (2009) [17]. The number of peaks detecting when running either MACS or BCP in its conventional form, using the ChIP-seq sample as the test and the input sample as the control was determined. Then the two samples were inverted, using the input sample as the test and the ChIP-seq sample as the control, to define the number of negative peaks. The empirical FDR was computed as the negative peaks divided by the test peaks.

Motif matches were identified using the STORM software package available in the CREAD software suite [48], [49]. Significant matches to the CTCF motif (accession no. MA0139) in the JASPAR database [46] or either STAT1 motif (accession nos. M00224 and M00492) in the TRANSFAC database [47] had a p-value less than 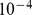. Peaks were evaluated in rank order (according to the enrichment score calculated by MACS or BCP) one at a time, and the cumulative motif occurrence rate (the number of peaks with at least one motif divided by the number of peaks evaluated) was tracked.

### Data description

We obtained publicly available datasets from the National Center for Biotechnology Information (NCBI) Gene Expression Omnibus (GEO, http://www.ncbi.nlm.nih.gov/geo/roadmap/epigenomics/). The following datasets were used: H3K36me3 (GSM521890), H3K36me3, replicate 2 (GSM521892), H3K27me3 (GSM469968), H3K27ac (GSM469966), H3K9ac (GSM469973), H3K4me3 (GSM469970), H3K9me3 (GSM469974), Input DNA for replicates 1 (GSM521926) and 2 (GSM521930), CTCF and input (GSM586887 and GSM586890), and STAT1 and input (GSM320736 and GSM320737). Functional annotations for genic regions were obtained from the UCSC Table Browser (GRCh37/hg19, February 2009, http://genome.ucsc.edu/) and intergenic region regions were further derived using the Galaxy Project data processing pipeline (http://galaxy.psu.edu).

### Availability

BCP software package is available for download at http://rulai.cshl.edu/BCP.

## Supporting Information

Text S1 Supporting materials for BCP.
(PDF)
