# Supplementary material for: Genome-Wide Localization of Protein-DNA Binding and Histone Modification by a Bayesian Change-Point Method with ChIP-seq Data
Source: PLoS Comput Biol. 2012 Jul 26;8(7):e1002613. doi: 10.1371/journal.pcbi.1002613 (PMC3406014; doi:10.1371/journal.pcbi.1002613)
Supplement: Text S1 — Supporting materials for BCP. (PDF) [file pcbi.1002613.s001.pdf]

Supporting Information File (Text S1)

Haipeng Xing, Yifan Mo, Will Liao, Michael Q. Zhang

Bounded Complexity Mixture (*BCMIX*) Approximation

Although the weight (2) in manuscript uses a recursive updating procedure, the number of weights increases with t, resulting in unbounded computational complexity and memory requirements in estimating θ_t as t keeps increasing. To reduce the computational complexity, we use the *BCMIX* approximation proposed by Lai and Xing (2011), which use $M(p)$ components and the most recent $m(p)$ weights $p_{j,n}$ (with $n - m(p) < j \leq n$ and $m(p) < M(p)$) for the posterior density (1) in manuscript. In particular, let $\mathcal{K}_{t-1}(p)$ be the set of indices i for which $p_{i,t-1}$ is kept at stage $t-1$; thus, $\mathcal{K}_{t-1}(p) \supset \{t-1, , \cdots, t-m(p)\}$. At stage t, define $p^*_{i,t}$ as in (2) in manuscript for $i \in \{t\} \cup \mathcal{K}_{t-1}(p)$, and let i_t be the index not belonging to $\{t, \cdots, t-m(p)+1\}$ such that

$$p^*_{i_t,t} = \min\{p^*_{j,t} : j \in \mathcal{K}_{t-1}(p) \quad \text{and} \quad j \leq t - m(p)\},$$

choosing i_t to be the minimizer farthest from t if the above set has two or more minimizers. Define $\mathcal{K}_t(p) = \{t\} \cup (\mathcal{K}_{t-1}(p) - \{i_t\})$, and let

$$p_{i,t} = \Big(p^*_{i,t} \Big/ \sum_{j \in \mathcal{K}_t(p)} p^*_{j,t}\Big), \quad i \in \mathcal{K}_t(p).$$

Similarly, to obtain a BCMIX approximation to (3) in manuscript, let $\widetilde{\mathcal{K}}_{t+1}(p)$ denote the set of indices j for which $q_{j,t+1}$ in (4) in manuscript is kept at stage $t+1$; thus, $\widetilde{\mathcal{K}}_{t+1}(p) \supset \{t+1, , \cdots, t+m\}$. At stage t, define $q^*_{j,t}$ as in (4) in manuscript for $j \in \{t\} \cup \widetilde{\mathcal{K}}_{t+1}(p)$, and let j_t be the index not belonging to $\{t, \cdots, t+m(p)-1\}$ such that

$$q^*_{j_t,t} = \min\{q^*_{j,t} : j \in \widetilde{\mathcal{K}}_{t+1}(p) \quad \text{and} \quad j \geq t + m(p)\},$$

choosing j_t to be the minimizer farthest from t if the above set has two or more minimizers. Define $\widetilde{\mathcal{K}}_t(p) = \{t\} \cup (\widetilde{\mathcal{K}}_t(p) - \{j_t\})$ and let $q_{j,t} = \Big(q^*_{j,t} \Big/ \sum_{j \in \widetilde{\mathcal{K}}_t(p)} q^*_{j,t}\Big)$, $j \in \widetilde{\mathcal{K}}_t(p)$, which yields a BCMIX approximation to the density $f(\theta_t | \mathcal{Y}_{t+1,n})$.

The BCMIX approximation to the smoother can be obtained by combining the forward and backward BCMIX filters via Bayes' theorem:

$$f(\theta_t | \mathcal{Y}_n) \approx \sum_{i \in \mathcal{K}_t(p),\ j \in \widetilde{\mathcal{K}}_{t+1}(p)} \gamma_{ijt} \pi(\theta_t; a_0 + j - i + 1, \bar{\mathbf{Y}}_{i,j}),$$

in which $\gamma_{ijt} = \gamma^*_{ijt}/\widetilde{P}_t$, $\widetilde{P}_t = p + \sum_{1 \le t \le n, i \in \mathcal{K}_t(p), j \in \widetilde{\mathcal{K}}_{t+1}(p)} \gamma^*_{ijt}$, and β^*_{ijt} given by (**??**) for $i \in \mathcal{K}_t(p)$ and $j \in \widetilde{\mathcal{K}}_{t+1}(p)$. The BCMIX approximation to $E(\theta_t|\mathcal{Y}_n)$ is therefore

$$\widehat{\theta}_t = \sum_{i \in \mathcal{K}_t(p),\ j \in \widetilde{\mathcal{K}}_{t+1}(p)} \gamma_{ijt}\alpha_{ij}\beta_{ij}.$$

The BCMIX approximation is accurate as it converges to the true θ_t when the sample size become larger; see the discussion on the efficiency and convergence of the BCMIX approximation in Lai and Xing (2011). Note that the BCMIX approximation $\widehat{\theta}_t$ reduce the computational complexity of estimating $\{\theta_t\}_{1 \le t \le n}$ from $O(n^3)$ to $O(n)$, which greatly reduces computational time and memory requirement in practice and are much faster than other methods in the literature.

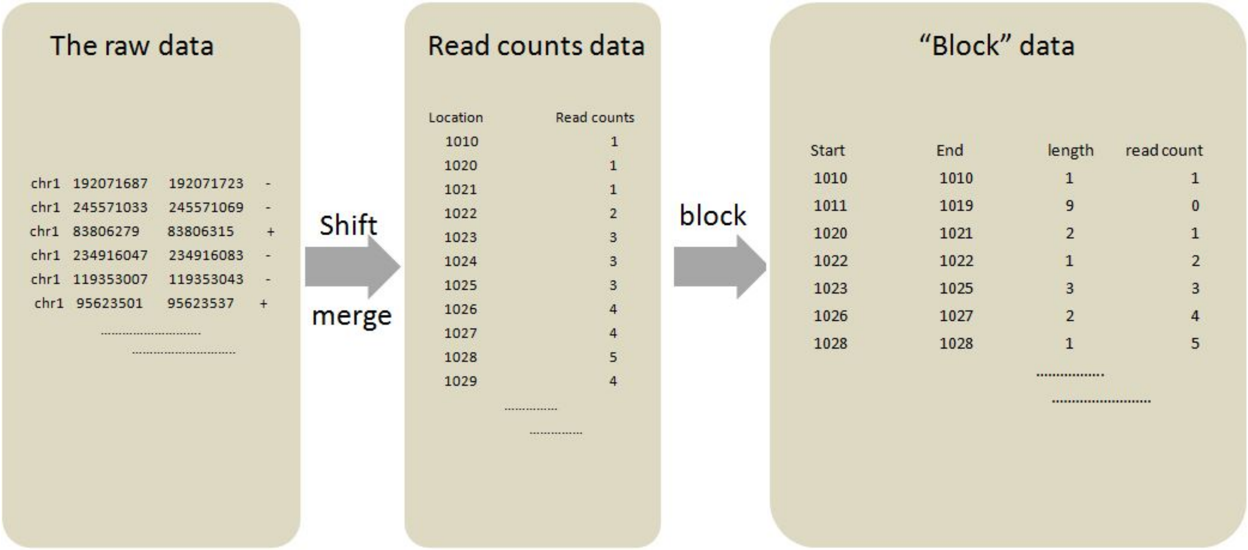

Figure S1. Pre-processing data for transcription factor case

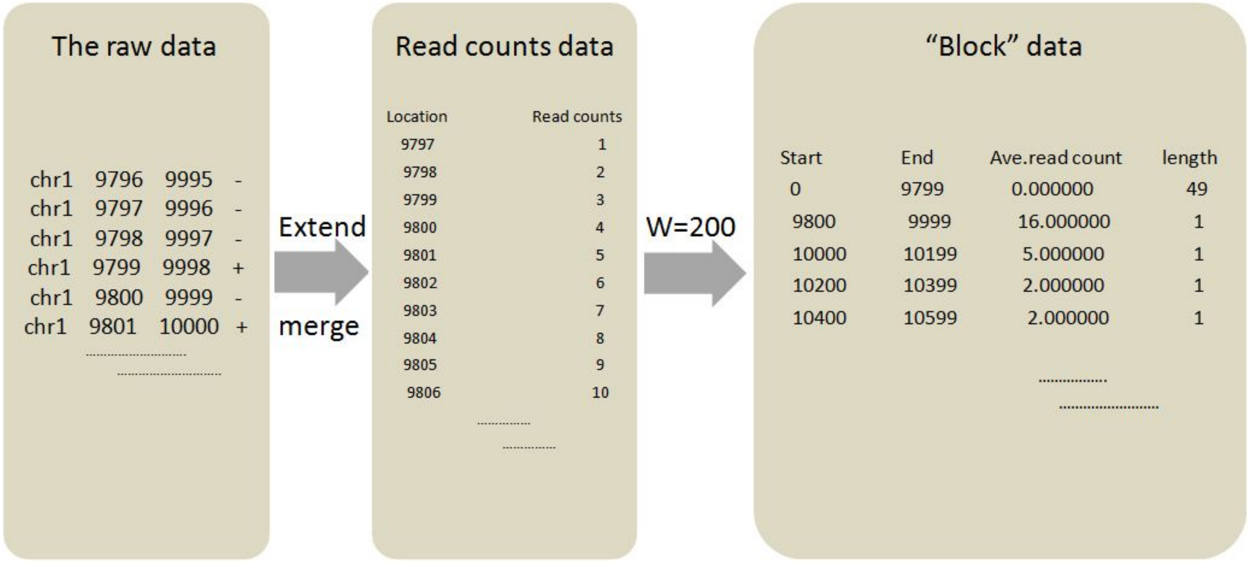

Figure S2. Pre-processing data for histone modification case with window size200*bp*.

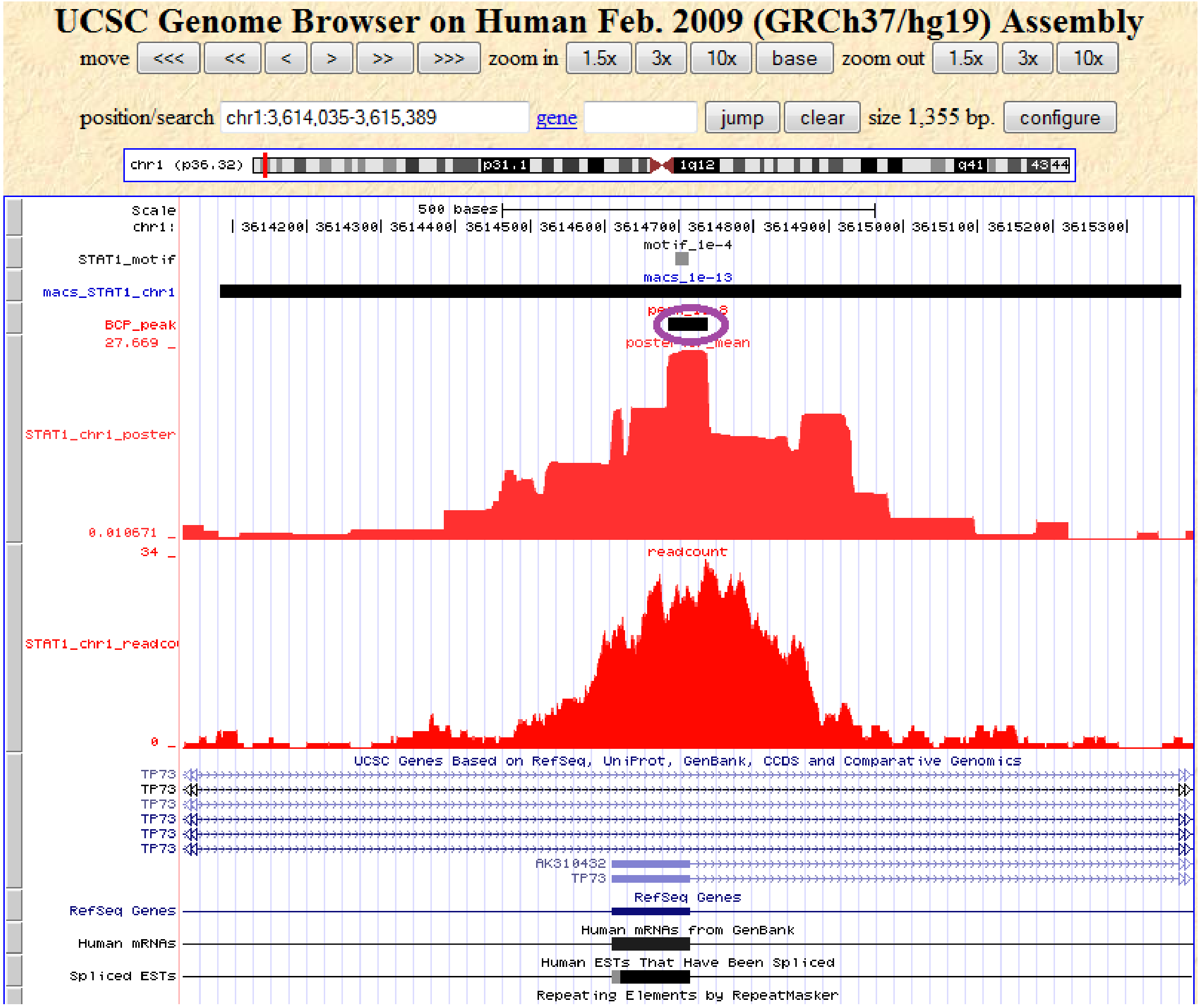

Figure S3. Choosing the most enrichment area as the candidate peak for TFBS indicated by the purple circle.

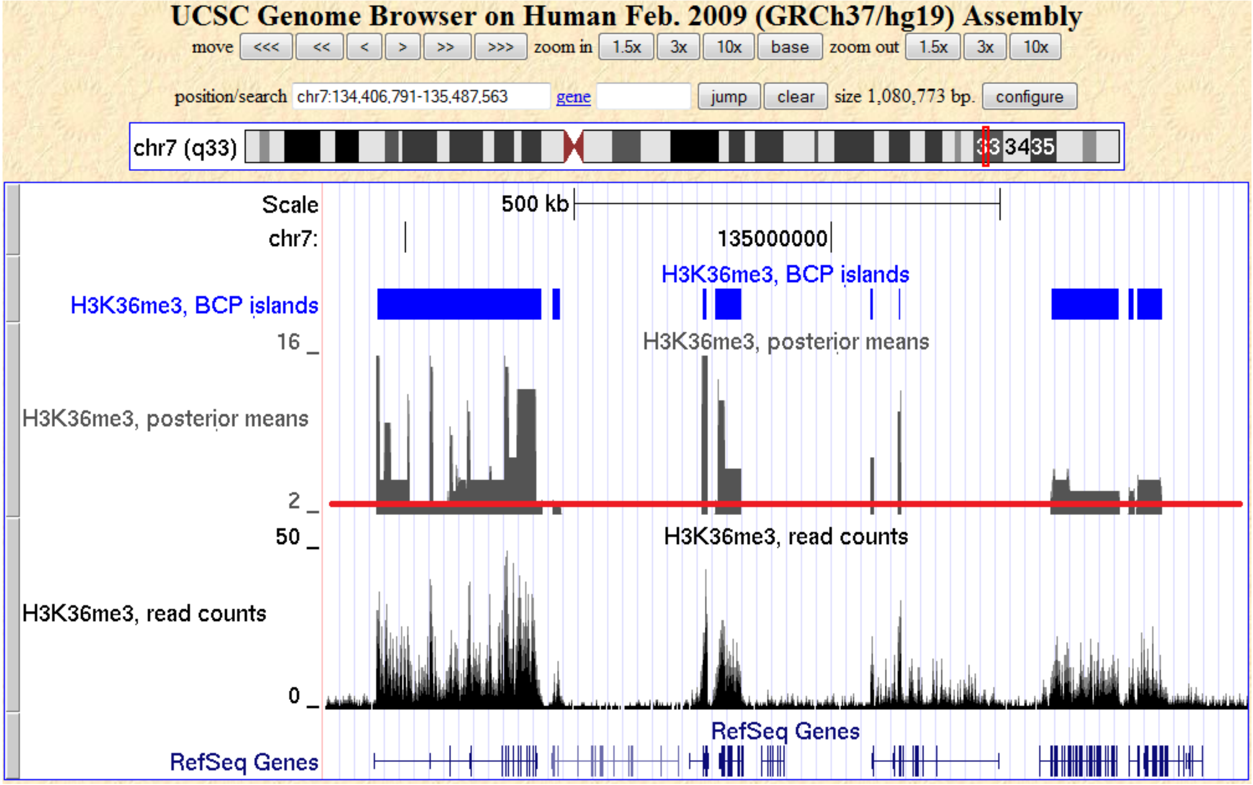

Figure S4. Choosing the candidate segments for HM.The red line is the threshold,regions beyond the red line will generate candidate segments.

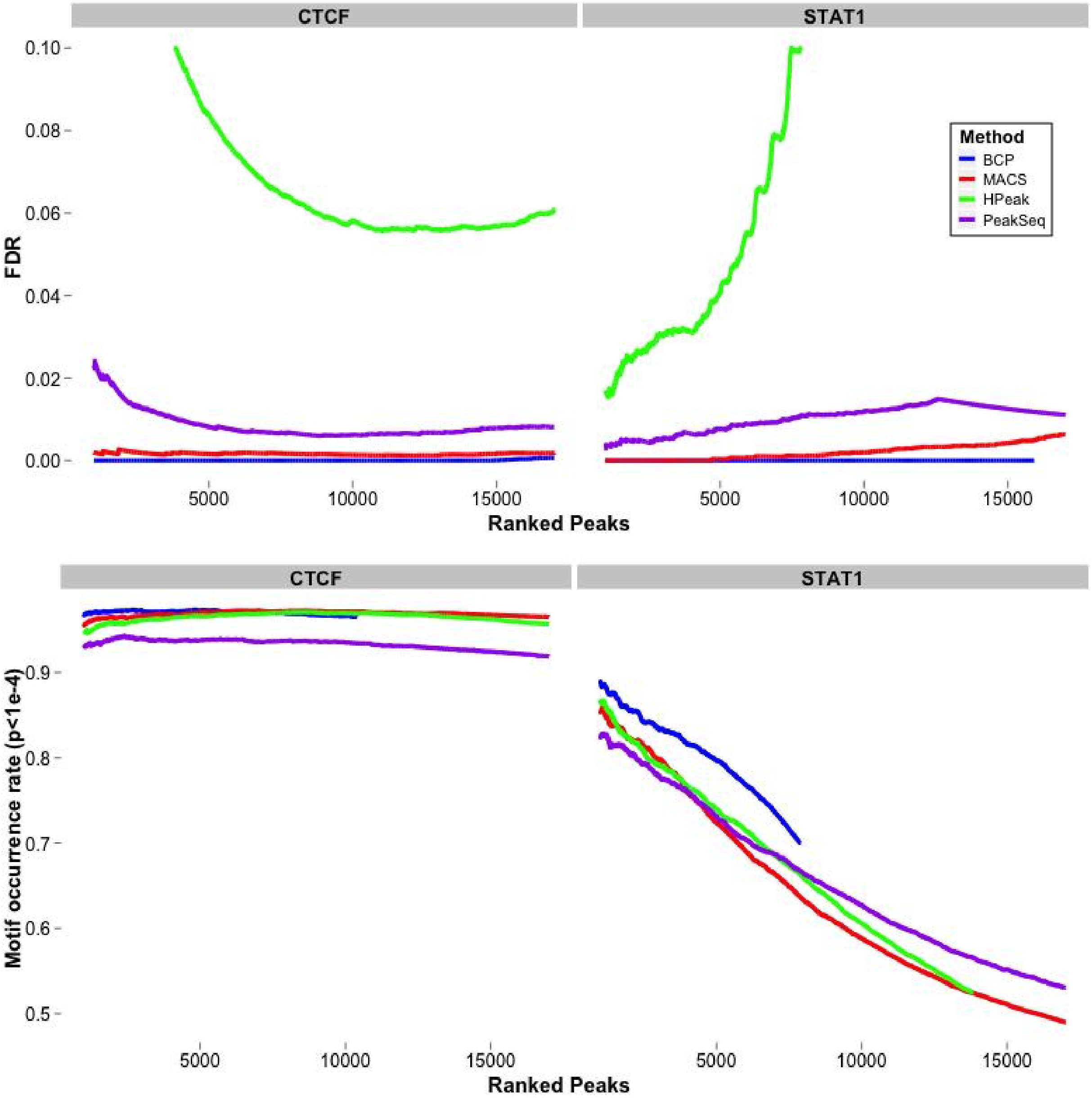

Figure S5. Along with diffuse histone data, BCP showed strong performance in punctate transcription factor ChIP-seq data. Comparing to MACS, HPeak, and PeakSeq, peak-calling algorithms designed with punctate peaks in mind, BCP shows a comparable or improved false-discovery rate (FDR) and rate of motif occurrence within called peaks. Peaks are ranked according to p-value.

Table S1. Island coverage (the fraction of aligned reads falling within islands of enrichment) was used to neutralize parameter-dependent fluctuation so BCP, MACS and SICER could be compared fairly. MACS displayed very low island coverage across all p value thresholds suggesting poor performance, as expected. BCP "threshold" generically describes thresholds used to identify regions of enrichment from background based on posterior means—ranging from the 50th to the 90th-quantile read count value based on the a Poisson distribution with mean determined from the whole data set. BCP Islands were routinely larger than MACS as well as SICER—even at similar island coverage in both H3K27me3 and H3K36me3 data sets. Given MACS was not designed for identifying broad regions of enrichment, it was not surprising to see it did not perform well in this test.

		**H3K27me3**			**H3K36me3**		
	parameters	Avg. island size (kb)	Genome coverage	Island coverage	Avg. island size (kb)	Genome coverage	Island coverage
**BCP**	threshold 1	49.2	0.210	0.680	54.8	0.170	0.720
	threshold 2	41.8	0.190	0.680	48.0	0.160	0.710
	threshold 3	32.9	0.170	0.630	35.9	0.140	0.690
	threshold 4	26.8	0.140	0.600	28.5	0.120	0.660
	threshold 5	22.9	0.120	0.560	23.9	0.110	0.630
**MACS**	$p < 1e-1$	1.7	0.132	0.130	2.4	0.107	0.106
	$p < 1e-2$	1.9	0.097	0.096	2.5	0.086	0.085
	$p < 1e-4$	2.1	0.063	0.063	2.6	0.067	0.066
	$p < 1e-6$	2.2	0.046	0.046	2.4	0.054	0.053
**SICER**	W200-G200	2.5	0.080	0.520	2.0	0.060	0.540
	W200-G400	4.2	0.100	0.550	3.2	0.070	0.570
	W200-G800	6.0	0.090	0.520	8.7	0.110	0.660
	W400-G400	4.7	0.103	0.566	6.8	0.070	0.651
	W400-G800	7.5	0.119	0.590	10.7	0.110	0.667
	W400-G1200	10.4	0.131	0.608	14.8	0.060	0.678

Table S2. Overlaps Ratio Here we give more parameter settings and corresponding association of Table 1. Additionally, BCP has a p-value threshold for calling significant islands—modeling the number of ChIP reads within a segment on a Poisson distribution with a mean derived from control data set. Scaling this parameter does not substantially affect island detection in relation to varying width and gap parameters in SICER. MACS did not perform well as was excluded from the remainder of the diffuse island analysis.

	Parameters	Average island size (kb)	Island coverage	Fraction of gene covered by island	Island covered by intergenic	Island covered by H3K27me3	Rep. 1 covered by rep. 2	Rep. 2 covered by rep. 1
**BCP**	$p < 1e-5$	25.8	0.629	0.497	0.089	0.019	0.851	0.805
	$p < 5e-5$	25.5	0.630	0.496	0.089	0.019	0.852	0.804
	$p < 1e-4$	25.3	0.630	0.496	0.089	0.019	0.852	0.804
	$p < 5e-4$	24.9	0.631	0.494	0.090	0.020	0.852	0.803
	$p < 1e-3$	24.7	0.631	0.494	0.090	0.020	0.852	0.803
	$p < 5e-3$	24.1	0.632	0.493	0.090	0.020	0.853	0.803
	$p < 1e-2$	23.9	0.632	0.492	0.090	0.021	0.853	0.802
	$p < 5e-2$	23.3	0.633	0.492	0.091	0.022	0.852	0.801
	$p < 1e-1$	23.1	0.634	0.491	0.091	0.022	0.852	0.800
**MACS**	$p < 1e-1$	2.4	0.130	0.337	0.908	0.025	0.726	0.713
	$p < 1e-2$	2.5	0.096	0.329	0.923	0.011	0.787	0.696
	$p < 1e-4$	2.6	0.063	0.285	0.932	0.005	0.834	0.618
	$p < 1e-6$	2.4	0.046	0.246	0.935	0.002	0.848	0.571
**SICER**	W200-G200	2.7	0.616	0.323	0.085	0.021	0.689	0.805
	W200-G400	4.5	0.636	0.370	0.088	0.025	0.736	0.814
	W200-G800	8.7	0.661	0.437	0.094	0.032	0.800	0.818
	W50-G200	1.6	0.584	0.268	0.081	0.015	0.522	0.815
	W50-G400	4.1	0.621	0.356	0.086	0.022	0.606	0.842
	W50-G800	11.9	0.656	0.469	0.096	0.031	0.716	0.852
	W400-G800	6.8	0.667	0.276	0.095	0.032	0.796	0.818
	W400-G1200	10.7	0.678	0.295	0.098	0.036	0.835	0.816
